# Interactions between lean management and the psychosocial work environment in a hospital setting – a multi-method study

**DOI:** 10.1186/1472-6963-14-480

**Published:** 2014-10-22

**Authors:** Waqar Ulhassan, Ulrica von Thiele Schwarz, Johan Thor, Hugo Westerlund

**Affiliations:** Department of Learning, Informatics, Management and Ethics, Medical Management Centre, Karolinska Institutet, Plan 5, Tomtebodavägen 18A, SE-17165 Stockholm, Sweden; Department of Psychology, Stockholm University, Stockholm, Sweden; Jönköping Academy for Improvement of Health and Welfare, Jönköping University, Jönköping, Sweden; Stress Research Institute, Stockholm University, Stockholm, Sweden

**Keywords:** COPSOQ, Stress, Employee involvement, Nurses, Sweden

## Abstract

**Background:**

As health care struggles to meet increasing demands with limited resources, Lean has become a popular management approach. It has mainly been studied in relation to health care performance. The empirical evidence as to how Lean affects the psychosocial work environment has been contradictory. This study aims to study the interaction between Lean and the psychosocial work environment using a comprehensive model that takes Lean implementation information, as well as Lean theory and the particular context into consideration.

**Methods:**

The psychosocial work environment was measured twice with the Copenhagen Psychosocial Questionnaire (COPSOQ) employee survey during Lean implementations on May-June 2010 (T1) (n = 129) and November-December 2011 (T2) (n = 131) at three units (an Emergency Department (ED), Ward-I and Ward-II). Information based on qualitative data analysis of the Lean implementations and context from a previous paper was used to predict expected change patterns in the psychosocial work environment from T1 to T2 and subsequently compared with COPSOQ-data through linear regression analysis.

**Results:**

Between T1 and T2, qualitative information showed a well-organized and steady Lean implementation on Ward-I with active employee participation, a partial Lean implementation on Ward-II with employees not seeing a clear need for such an intervention, and deterioration in already implemented Lean activities at ED, due to the declining interest of top management. Quantitative data analysis showed a significant relation between the expected and actual results regarding changes in the psychosocial work environment. Ward-I showed major improvements especially related to job control and social support, ED showed a major decline with some exceptions while Ward-II also showed improvements similar to Ward-I.

**Conclusions:**

The results suggest that Lean may have a positive impact on the psychosocial work environment given that it is properly implemented. Also, the psychosocial work environment may even deteriorate if Lean work deteriorates after implementation. Employee managers and researchers should note the importance of employee involvement in the change process. Employee involvement may minimize the intervention’s harmful effects on psychosocial work factors. We also found that a multi-method may be suitable for investigating relations between Lean and the psychosocial work environment.

**Electronic supplementary material:**

The online version of this article (doi:10.1186/1472-6963-14-480) contains supplementary material, which is available to authorized users.

## Background

Lean Management, Lean Thinking or Japanese Production Management (below called Lean) was introduced by Toyota to improve both efficiency and quality on production lines. This was done by identifying quality problems early and eliminating wasteful aspects like unproductive waiting time and unnecessarily large stocks [1]. Following this, Lean has been widely adopted across the world in manufacturing and later also in health care [2–4]. When the effects of Lean have been researched, the focus has often been on process outcomes [3]. However, since Lean often involves changes in fundamental aspects of work such as work process and work structure [3], Lean is also likely to affect the working conditions of the employees [5–7]. In line with this, Lean has been criticized as being a way in which management increases the demands on the employees [8]. However, in research on psychosocial work aspects in relation to Lean to date, both positive and negative impacts have been seen [8]. For example, when Lean leads to downsizing [9], the effects are likely to be very different from those expected from an integrated Lean approach in line with the broader socio-technical environment [10]. The negative associations between Lean and psychosocial work environments have been reported to be particularly pronounced in the manufacturing industry, while a mix of positive and negative correlations have been found in other settings including health care [11]. With similar findings, a recent review suggested a move from a cause-and-effect model to a more comprehensive model that sees Lean as an open and ambiguous concept which may have either positive or negative effects, or even both, depending on the actual Lean intervention implementation [8]. That review also states that research into the effect of Lean on the working environment is still limited outside of manufacturing industry, and suggests that the effects of Lean should be studied not only based on the concept itself but also on the Lean practice and the context into which it is introduced. In line with this, one large scale industrial study [5] reported that the relationship between Lean and stress differed depending on how the psychosocial working conditions had changed as a result of Lean. In that study, control over work speed and work flow, employee involvement and influence on the change process, team-based organization and management support were related to reduced stress while an increase in work speed, removal of resources, increased working hours and employees’ feeling blamed for defects were related to increased stress levels. Another study showed that the active participation of employees in change processes was strongly associated with psychosocial aspects related to job control (influence, possibilities for development, meaning of work, freedom at work and commitment), social support (supervisors’ and colleagues’ support and quality of leadership, sense of community, role conflict and role clarity) and rewards (esteem) [12]. In fact, an employee-centered Lean intervention implemented with good employee participation has been considered as the most important means of preventing negative effects on the psychosocial work environment [3, 8, 12]. In summary, further research is needed into the interaction between work organization interventions such as Lean and the psychosocial work environment, in order to improve such interventions’ design so that the psychosocial work environment is improved [6]. This research is particularly needed in health care, given the steady growth of Lean application in this area [13].

### Theoretical background

The psychosocial work environment is a complex concept that involves factors relating to the objective situation, the individual’s perception of the situation and the situation’s consequences, e.g. ill-health. The demand-control model (DCM), the effort-reward-imbalance model (ERI) and the job demands-resources model (JD-R) are all theoretical models that have become essential for researching psychosocial aspects of work [14]. The DCM, devised by Karasek, describes work stress as a consequence of an imbalance between demands at work and job autonomy [15]. An important point in this model is that a high degree of job autonomy and influence at work may prevent work stress, even when job demands are high. From another perspective, the ERI describes work stress as a consequence of an imbalance between efforts and rewards [16]. The JD-R, devised by Demerouti and colleagues, states that although the specific risk factors differ between occupations, these risk factors can be classified in two broad categories: job demands and job resources [17]. The model also states that these two factors relate differently to job motivation and job strain and it is highlighted that the model covers both positive and negative aspects of the psychosocial work environment [18].

In light of the uncertainty regarding the impact on staff when applying Lean in health care, this comparative longitudinal study examines changes in the psychosocial work environment among hospital staff during Lean-inspired changes. Rather than finding a causal relation, the focus is on capturing a “Lean footprint” on the psychosocial work environment using 19 subscales from the Copenhagen Psychosocial Questionnaire (COPSOQ), including most aspects covered by the major occupational health theories described above [19].

## Methods

### Study design and procedures

This is a longitudinal multi-method observational study of the introduction of Lean, at the Emergency Department (ED) and two inpatient cardiac wards (Ward-I and Ward-II) at Danderyd hospital, an acute care hospital in suburban Stockholm Sweden, and its consequences for the psychosocial work environment. Lean implementation started in November 2008 at the ED and January 2010 on Ward-I and Ward-II. Data was collected from nurses and nurse aides working in the three units (excluding staff on sick-leave or maternity leave). The data collection was done twice, ‘early and later’, during the Lean implementation process; first in May-June 2010 (T1) and secondly in November-December 2011 (T2). The same questionnaire was used both times; it included several COPSOQ scales [20] and demographic background questions. Therefore, all dimensions were assessed twice. For the data collection process, one person was assigned by the respective hospital departments as a facilitator in each unit. Prior to giving informed consent, the participants were given written and oral information about the study. The study was approved by the Regional Ethical Review Board in Stockholm.

### Description of the sample

The sample characteristics for each unit are given in Table 1. All the nurses and nurse aides at the three settings were included in the survey. In the T1 sample, the number of returned questionnaires was 129 (58%); 1 was returned without complete information; therefore, 128 were retained in the analyses. In the T2 sample, the number of returned questionnaires was 131 (64%); 3 were returned without complete information; therefore, 128 were retained in the analyses. 55 of the respondents at T2 (43%) had also responded at T1. The T1 and T2 samples showed no significant difference in demographic characteristics.

**Table 1:** **Characteristics of the sample for each unit**

	Ward-I		ED		Ward-II		Overall	
Source	T1	T2	T1	T2	T1	T2	T1	T2
Response rate	68	64	56	64	59	54	58	62
Same respondents^a^		50		47		36		43
Women	82	63	84	83	94	92	86	81
Age								
Under 30	30	13	30	10	28	24	29	13
31-45	47	56	55	67	38	44	50	61
Over 45	23	31	15	23	34	32	21	26
Education								
School/College	35	13	55	34	28	24	46	30
University	65	87	45	66	72	76	54	70
Profession								
Nurse aid	41	31	59	40	41	44	52	40
Nurse	59	69	41	60	59	56	48	60
Experience								
< 5 year	88	75	54	46	47	60	57	52
5 – 20 years	12	13	39	51	41	24	36	41
> 20 years	0	12	7	3	13	16	7	7

Note. Values are expressed as a percentage of the total number of respondents.

^a^Values only shown for T2 as a percentage of respondents who responded at both T1 and T2 to the total number of respondents at T2.

### Measure – the research instrument

The COPSOQ is a comprehensive questionnaire which includes several psychosocial aspects covered by major occupational health theories [19]. The COPSOQ authors intentionally made it “theory-based without being based on one specific theory” and justified this by arguing that no single theory encompasses all the important psychosocial work factors [19]. Only one psychosocial factor, i.e. ‘rewards’, related to the ERI model, is not fully captured by COPSOQ, as only one of its three components, i.e. recognition, is included [20]. Thus, several factors need to be considered in psychosocial work environment studies in order to capture this range of important factors.

The Swedish version of the Copenhagen Psychosocial Questionnaire (COPSOQ-II) [20] was used. Starting with the long-version, the questionnaire was shortened and only the scales deemed relevant to the intervention were included. Out of 41 scales, 19 were used. These consisted of 5 full scales and 14 partial scales, divided into four domains (see Table 2). Out of the total 45 items, 13 items were scored from 1 (Always) to 5 (Never), 5 items were scored from 1 (Always) to 6 (Not related) and 27 items were scored from 1 (To a very high degree) to 5 (Almost not at all). Following the scoring procedure suggested by the COPSOQ authors [19], each scale was summed up and rescaled from 0 to 100, with 100 representing the highest degree of the measured psychosocial factor and 0 representing the lowest. Scoring follows the label of the scale, e.g. a higher score on the role clarity scale means more role clarity, and a lower score on the emotional demands scale means fewer emotional demands and so on. Therefore, depending on the type of scale, a high score can be either undesirable (e.g. Emotional demands) or desirable (e.g. Role clarity). The COPSOQ has been subjected to a number of statistical tests regarding its reliability and validity [20, 21] and shown to have appropriate psychometric properties.

**Table 2:** **Copenhagen Psychosocial Questionnaire domains, scales and sample items used in this study**

Domain	Scale name	Sample items
Demands at work	Quantitative demands	Do you get behind with your work?
	Tempo at work	Do you have to work very fast?
	Cognitive demands	Do you have to keep your eyes on lots of things while you work?
	Emotional demands	Does your work put you in emotionally disturbing situations?
	Demands for hiding emotions*	Does your work require that you hide your feelings?
Work organization and job content	Influence at work	Can you influence the amount of work assigned to you?
	Possibilities for development	Does your work require you to take the initiative?
	Meaning of work	Do you feel that the work you do is important?
	Commitment to the workplace	Do you feel that your place of work is of great importance to you?
	Rewards at work	Is your work recognised and appreciated by the management?
Interpersonal relations and leadership	Predictability*	Do you receive all the information you need in order to do your work well?
	Role clarity	Does your work have clear objectives?
	Role conflicts*	Are contradictory demands placed on you at work?
	Social support from colleagues	How often do you get help and support from your colleagues?
	Social support from supervisors	How often is your nearest superior willing to listen to your problems at work?
	Social community at work*	Is there a good atmosphere between you and your colleagues?
Values at the workplace	Horizontal trust*	Do the employees in general trust each other?
	Vertical trust	Does the management trust the employees to do their work well?
	Justice and respect	Are conflicts resolved in a fair way?

Note: Scales denoted by an asterisk (*) were used in full while others were used partially.

### Analysis

**Figure 1:**
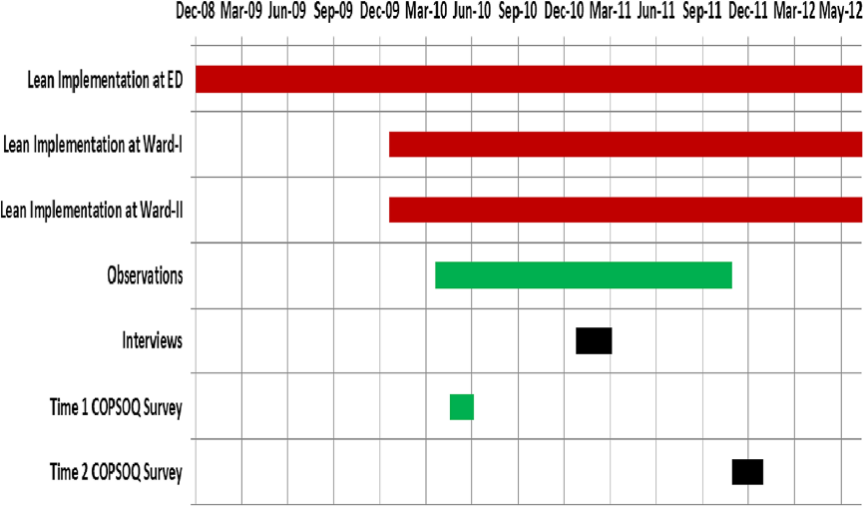
**Timeline for Lean intervention and data collection at three settings.** Red bars denote Lean intervention, black bars denote interviews, green bars denote observations and blue bars denote surveys.

To avoid post-hoc explanations, we predicted expected changes for each COPSOQ subscale before analysis, based on what was known about the context, planning and execution of the Lean implementation for each of the three units. This information was based on qualitative data analyzed and presented in a previous study [22]. According to that information, Ward-I had started to implement Lean a few months before T1 and the execution of the Lean implementation ran quite smoothly between T1 and T2, with leadership support and high employee involvement. ED was well underway in implementing Lean at T1, and was achieving positive process outcomes. However, after T1, the Lean routines gradually deteriorated until T2, when hardly any Lean-related changes remained, except for teamwork. Concurrently, the ED experienced organizational turmoil with big managerial changes and unwillingness among physicians to part take in Lean improvement work. Ward-II started implementing Lean almost at T1 but had only implemented it partially by T2. One of the main reasons for this was that employees did not experience any real need for process improvements in their work. Table 3 gives an overview of the different Lean intervention parts implemented at the three units. Figure 1 shows a timeline of Lean intervention and data collection points for this study. Before analysis, in the light of our synthesis of Lean literature on the psychosocial work environment [1, 5, 8, 23–25], we also predicted that some of the scales were most likely to be affected by Lean implementation. These scales (below called ‘most relevant to Lean’) were: Quantitative demands, Cognitive demands, Influence at work, Meaning of work, Commitment to the workplace, Predictability, Role clarity and Vertical trust. Considering the four domains, we predicted that the two domains ‘Work Organization and job content’ and ‘Interpersonal relations and leadership’ are most responsive to Lean. Again, this was done in order to avoid relying on post-hoc explanations for the findings.

**Table 3:** **An overview of the Lean intervention parts implemented at the three units**

Lean intervention	Ward-I	Ward-II	ED
**Education/Training**	2 days training for the whole staff by external consultant	2 days training for the whole staff by external consultant	Only Lean coaches were trained by the hospital’s Quality Development Group
**5S**	Executed with keen employee involvement	Executed with the support of Lean coaches	Executed with the support of Lean coaches
**Value Stream Map**	Executed by Lean coaches including employee discussions	Executed by Lean coaches	Executed by the flow group
**Continuous Improvement & Visual Management**	Executed through employee discussions and sustained	Started by Lean coaches but couldn’t be sustained	Planned by the flow group but couldn’t be implemented
**Work Redesign**	A joint working station for physicians and nurses, nurse aides equipped with trolleys having laptops, colored magnets for patient status, patients seen one by one.	One working station for physicians and nurses, nurse aides equipped with trolleys having laptops, colored magnets for patient status, patients seen one by one.	One working station for physicians and nurses, one part-time junior physician now full-time, ECG machine stationed in preliminary care room, heart coordinator to admit patients from ED to wards
**Teamwork**	2 care teams; in each team, physicians and nurses started working in pairs	3 care teams; in each team, physicians and nurses started working in pairs	Team for preliminary care consisting of a nurse and a junior physician led by a specialist

The expected changes for each unit were predicted after discussion among all the authors in a one day interactive session. For each scale, we predicted the expected change as positive, negative or null, where null denoted no change, defined as a change between +1.5 and -1.5 in scale score value. Then, we compared the actual outcome with these expected changes, and evaluated the changes found in the scales. The survey data were analyzed using SPSS (Statistical Package for Social Sciences, version 19). Data were analyzed using parametric statistics to identify the change patterns between T1 and T2 in different psychosocial factors at the three units. Firstly, individual means for actual changes were computed for T1 and T2 for all the scales. After Z-score transformations, differential scores were obtained by subtracting T1 scores from T2 scores. To avoid issues relating to an inflated number of statistical tests and to make the most of the design of the study, the focus was on the pattern of changes found in different aspects of psychosocial factors instead of simply comparing pre- to post-change separately for each scale. Thus, the overall pattern of net scores for each group was compared with the respective expected pattern through linear regression analysis.

## Results

Table 4 describes our predictions for changes in all the scales in all three settings along with motivations behind it. The results of regression analysis of expected versus actual changes were found to be significant in all three units (ED: F(1,17) = 5.88; *p* = .03; Beta = .507; Ward-II: F(1,17) = 7.68; *p* = .01; Beta = .558; Ward-I: F(1,17) = 5.50; *p* = .03; Beta = .494). Table 5 shows the COSPSOQ scales scores for T1 and T2 in three settings.

**Table 4:** **Changes expected in Copenhagen Psychosocial Questionnaire scales in three units**

	Scale	Change	Motivation
**Ward-I**	Quantitative Demands	-1	Better work design due to Lean intervention
	Tempo	-1	-do-
	Cognitive Demands	+1	CI work and use of VM
	Emotional Demands	0	No related to Lean or some other change
	Demands for Hiding Emotions	0	Not related to Lean intervention at Ward-I
	Influence at Work	+1	Lean with CI activity
	Possibilities for Development	+1	-do-
	Meaning of Work	+1	Steady Lean intervention with CI
	Commitment to Workplace	+1	-do-
	Predictability	+1	CI with VM
	Rewards at Work	+1	Lean with a supportive leadership
	Role Clarity	+1	Improved work organization as a result of Lean
	Role Conflicts	-1	Lean generally
	Social support from Colleagues	+1	Teamwork, CI and new seating plan
	Social support from Supervisors	+1	Supportive leadership
	Social Community at Work	+1	Steady Lean having teamwork
	Horizontal Trust	0	Already good enough at T1
	Vertical Trust	0	-do-
	Justice and Respect	+1	Physicians and nurses working in pairs may lead to feeling of fair work distribution
**Ward-II**	Quantitative Demands	-1	High value at T1 due to high patient volume and Lean work
	Tempo	-1	-do-
	Cognitive Demands	-1	CI and VM being tried at T1 but couldn’t work
	Emotional Demands	+1	Bad Lean perception increased dissatisfaction
	Demands for Hiding Emotions	0	Not related to Lean intervention at Ward-II
	Influence at Work	+1	VSM, work redesign and teamwork
	Possibilities for Development	0	No CI activity
	Meaning of Work	0	No CI activity mean no employee involvement
	Commitment to Workplace	+1	High turnover implies very low value at T1
	Predictability	0	Limited information dissemination as no VM
	Rewards at Work	0	Not related to Lean intervention at Ward-II
	Role Clarity	0	No CI to make roles clearer
	Role Conflicts	0	No other changes to increase role conflicts
	Social support from Colleagues	+1	Teamwork and new seating plan
	Social support from Supervisors	+1	Staff welcomed leadership change before T2
	Social Community at Work	+1	Teamwork and new seating plan
	Horizontal Trust	0	The scale item regarding management will cancel the effect of teamwork
	Vertical Trust	0	Distrust at T1due to Lean likely to be cancelled by trust in new leadership at T2
	Justice and Respect	+1	Physicians and nurses working in pairs may lead to feeling of fair work distribution
**ED**	Quantitative Demands	+1	Deterioration of Lean
	Tempo	+1	-do-
	Cognitive Demands	-1	Morning meetings with whiteboard being held at T1 but abandoned till T2
	Emotional Demands	0	Not related to Lean intervention at ED
	Demands for Hiding Emotions	0	-do-
	Influence at Work	0	Likely poor at T1 and remain poor at T2
	Possibilities for Development	-1	Deterioration of Lean
	Meaning of Work	-1	-do-
	Commitment to Workplace	-1	-do-
	Predictability	0	Not related to Lean intervention at ED
	Rewards at Work	-1	Deterioration of Lean
	Role Clarity	-1	-do-
	Role Conflicts	+1	-do-
	Social support from Colleagues	-1	Less teamwork as morning meetings abandoned
	Social support from Supervisors	-1	Withering leadership
	Social Community at Work	-1	Less teamwork
	Horizontal Trust	0	No Lean or other changes likely to change this
	Vertical Trust	-1	Deterioration of Lean
	Justice and Respect	0	-do-

**Table 5:** **Changes in Copenhagen Psychosocial Questionnaire scales in the three study units between T1 and T2**

Scale	ED				Ward-I				Ward-II			
	T1 (n =80)	T2 (n =87)	Diff	Hyp	T1 (n =17)	T2 (n =16)	Diff	Hyp	T1 (n = 32)	T2 (n = 25)	Diff	Hyp
Quantitative demands	44.9 (15.8)	52.6 (16.4)	7.7	1	38.2 (15.0)	36.7 (15.3)	-1.6	-1	45.7 (21.0)	40.5 (15.8)	-5.2	-1
Tempo at work	75.2 (13.4)	74.0 (15.8)	-1.2	1	59.6 (13.6)	64.2 (18.8)	4.6	-1	70.3 (18.7)	64.1 (12.9)	-6.3	-1
Cognitive demands	78.5 (12.4)	69.4 (18.7)	-9.1	-1	66.7 (16.4)	69.4 (14.3)	2.8	1	70.6 (13.0)	69.8 (18.4)	-0.8	-1
Emotional demands	52.4 (21.3)	60.1 (16.6)	7.7	0	55.1 (23.0)	56.3 (22.8)	1.1	0	50.0 (19.1)	55.0 (21.7)	5	1
Hiding emotions*	74.6 (13.0)	67.1 (16.5)	-7.5	0	71.1 (13.2)	70.6 (15.1)	-0.5	0	67.4 (12.6)	69.1 (14.8)	1.6	0
Influence at work	39.6 (18.7)	38.5 (17.7)	-1.1	0	40.4 (13.6)	50.0 (20.4)	9.6	1	33.6 (16.3)	45.5 (18.4)	11.9	1
Possibilities for development	72.9 (22.9)	70.8 (18.5)	-2.1	-1	65.4 (15.6)	66.7 (20.4)	1.2	1	69.1 (14.5)	72.4 (18.4)	3.3	0
Meaning of work	86.9 (15.2)	76.2 (16.5)	-10.7	-1	79.4 (13.2)	89.2 (10.4)	9.8	1	81.3 (13.5)	84.5 (11.6)	3.3	0
Commitment to the workplace	69.5 (20.2)	61.0 (20.1)	-8.4	-1	59.6 (21.4)	74.2 (16.0)	14.6	1	51.6 (19.5)	60.0 (17.7)	8.4	1
Rewards at work	62.2 (18.7)	60.4 (15.5)	-1.7	0	58.8 (20.6)	75.9 (16.6)	17.1	1	60.2 (18.9)	66.7 (12.6)	6.5	1
Predictability*	57.0 (15.7)	52.4 (15.7)	-4.5	-1	53.7 ( 9.6)	65.8 (16.7)	12.2	1	49.2 (13.8)	51.5 (19.2)	2.3	0
Role clarity	75.0 (14.7)	67.2 (15.9)	-7.8	-1	64.0 ( 9.8)	80.8 (10.4)	16.9	1	62.5 (15.9)	68.5 (14.5)	6	0
Role conflicts*	48.7 (13.5)	52.2 (13.0)	3.5	-1	41.9 ( 9.3)	46.3 (20.2)	4.3	1	48.8 (12.1)	54.1 ( 7.4)	5.2	0
Social support from colleagues	80.6 (10.5)	80.8 (13.3)	0.2	-1	73.1 (11.4)	79.3 ( 9.6)	6.2	1	76.5 (12.0)	74.2 (12.1)	-2.3	1
Social support from supervisors	56.2 (29.7)	62.1 (23.2)	5.9	-1	64.7 (22.6)	78.1 (16.8)	13.4	1	60.5 (21.3)	58.2 (20.2)	-2.4	1
Social community at work*	82.2 (8.7)	79.5 (11.5)	-2.7	-1	77.3 (14.2)	85.3 ( 8.4)	8.1	1	80.2 (12.0)	81.9 (10.1)	1.7	1
Horizontal trust*	32.0 (14.8)	43.0 (15.9)	11	0	29.9 (17.4)	26.1 (13.7)	-3.8	0	26.8 (14.2)	25.4 (14.2)	-1.4	0
Vertical trust	66.0 (17.0)	64.9 (18.0)	-1.0	-1	69.1 (12.6)	64.3 (21.3)	-4.8	0	56.3 (20.3)	63.5 (12.7)	7.3	0
Justice and respect	55.4 (15.6)	57.9 (17.4)	2.5	0	64.7 (14.1)	60.0 (18.4)	-4.7	1	51.6 (15.5)	63.5 (14.2)	12	1

Note. *Hyp* = Expected direction, *Diff* = Numerical difference between the T1 and T2 values of the relevant scale, n denotes the number of respondents in each setting at that time. Scales denoted by an asterisk (*) were used in full while others were used partially.

Since this study included one unit that successfully implemented Lean during the study period (Ward-I), one where previously implemented Lean changes deteriorated (the ED) and one which partially implemented Lean during the study period (Ward-II), we performed a comparison between the patterns for the three units (Additional file 1). Ward-I and the ED showed changes in different directions in 13 out of 19 scales; and Ward-II and the ED differed in 14 of the 19 scales. In contrast, Ward-I and Ward-II showed changes in the *same* direction in 12 of the 19 scales. Looking only at the domains ‘Work organization and job content’ and ‘Interpersonal relations and leadership’, as 5 out of the 8 scales expected beforehand to be most responsive to Lean were included in these two domains, 8 out of 11 scale changes were in the opposite direction when comparing ED and Ward-I and 10 out of 11 scale changes were in the opposite direction when comparing ED and Ward-II. Again, comparing Ward-I and Ward-II showed similar patterns: 9 out of 11 scale changes were in the same direction. In line with the Lean intervention outcomes in the three units, the results for ED are mostly contrary to the results for Ward-I while the results for Ward-II are mostly similar with Ward-I. This comparison is most pronounced for the domains ‘Work Organization and job content’ and ‘Interpersonal relations and leadership’. No clear pattern could be detected under the domain ‘Demands at work’ and ‘Values at the workplace’. Only one scale, ‘Horizontal trust’, changed contrary to the Lean intervention outcome, i.e. it deteriorated on Ward-I and Ward-II and improved at ED. Therefore, the overall change patterns for the three units are in line with the Lean intervention outcomes.

Comparing the expected and the actual changes, ten out of 19 actual changes were same as predicted for ED, 13 out of 19 actual changes were as predicted for Ward-I, and ten out of 19 actual changes were as predicted for Ward-II. Looking only at the domains ‘Work organization and job content’ and ‘Interpersonal relations and leadership’, as 5 out of the 8 scales deemed as most responsive to Lean were included in these, eight out of eleven actual changes were as predicted for ED, nine out of eleven actual changes were as predicted for Ward-I and only four out of eleven actual changes were as predicted for Ward-II. Looking at the results of four domains for all three units, nine out of 15 actual changes were as predicted for ‘Demands at work’, eleven out of 15 actual changes were as predicted for ‘Work organization and job content’, ten out of 18 actual changes were as predicted for ‘Interpersonal relations and leadership’ and only two out of nine actual changes were in the same direction as predicted for ‘Values at the workplace’. For Ward-I, there was only one scale (‘Justice and respect’) which was expected to increase but decreased and two scales (‘Tempo at work’ and ‘Role Conflicts’) which were expected to decrease but increased. There were four scales which were expected not to change, of which two decreased (‘Horizontal trust’ and ‘Vertical trust’) while the other two were almost unchanged. Importantly, there were only two scales (‘Possibilities for development’ and ‘Role Conflicts’) under the domains ‘Work organization and job content’ and ‘Interpersonal relations and leadership’ which did not change as expected but all the other scales changed as expected. For Ward-II and the ED, almost half of the scales changed as expected with varying domain wise results. With regards to the four domains, the ‘Work organization and job content’ domain showed maximum agreement between the expected and the actual changes. The ‘Interpersonal relations and leadership’ and the ‘Demands at work’ domains also showed good agreement but the quantity of change was higher in ‘Interpersonal relations and leadership’ than ‘Demands at work’. The ‘Quantitative demands’ and ‘Cognitive demands’ scales under the domain ‘Demands at work’, which were among the scales most relevant to Lean, changed as expected in all three units. The domain ‘Values at the workplace’ showed the least level of agreement and the scales are mostly not in line with what was expected. In summary, we can see the maximum agreement between the expected and the actual changes for Ward-I while for Ward-II and at ED, the agreement was medium.

**Figure 2:**
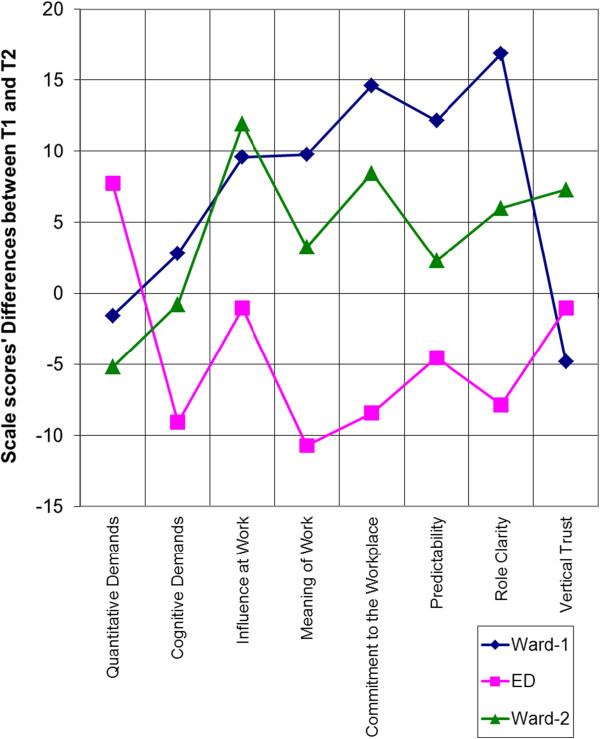
**Changes in scales relevant to Lean.** Graphical representation of changes found in only selected COPSOQ scales which were expected to be most responsive to Lean at three units.

Focusing particularly on those scales that were considered most relevant to Lean implementation before analysis, all the scales except the ‘Vertical Trust’ changed as expected for the ED and the Ward-I; however five out of eight scales changed as expected for Ward-II. The ‘Vertical Trust’ was the only scale which didn’t change as expected for all the three settings. Based on these results, Figure 2 shows that the Ward-I and Ward-II graphs are partly similar in pattern; however, the ED graph pattern is almost opposite in direction. The ‘Cognitive demands’ scale value at the Ward-II decreased, although a little, similar to the ED than the Ward-I. The ‘Vertical Trust’ scale value at the Ward-I decreased, similar to the ED than the Ward-II. With regards to the expected versus actual changes for these scales, most of the scales changed as expected. Only one scale (‘Vertical trust’) did not change as expected at any unit. Looking unit-wise at Ward-I and ED, all the scales changed as expected except ‘Vertical trust’. For Ward-II, only 4 out of the 8 scales changed as expected.

## Discussion

In this study, we aimed to investigate how implementing Lean in health care affects the psychosocial work environment. When we contrasted the relationship between Lean and the psychosocial work environment in three units which differed in their implementation of Lean, differing patterns emerged. This was particularly pronounced for the psychosocial work environment factors that were considered most relevant to a Lean intervention. The results suggest that when Lean was implemented (more) successfully, it was related to improvements in the domains ‘Work organization and job content’ and ‘Interpersonal relations and leadership’. Concurrently, in the unit where Lean deteriorated, the psychosocial work environment also deteriorated, while the unit that implemented Lean to a limited extent showed some limited improvements in the psychosocial work environment. In summary, this suggests that Lean, if implemented successfully, may be positively related to psychosocial work conditions.

Ward-I showed improvements in three of the four domains of the psychosocial work environment. The greatest improvements were seen in the domains ‘Work organization and job content’ and ‘Interpersonal relations and leadership’. This is in line with a previous study [5, 12] relating improvements in the same domains to active employee participation in Lean implementation, a supportive leadership and regular morning meetings, all of which were also the case on Ward-I. Overall, the ED showed deterioration in psychosocial work factors. This deterioration was more prominent in the ‘Work organization and job content’ and ‘Interpersonal relations and leadership’ domains. This seems to be in line with the deterioration of Lean activities between T1 and T2. For example, there were less frequent morning meetings and decreased employee participation at the ED which may have resulted in the deterioration of scales related to job control and social support [12]. Ward-II, like Ward-I, showed major improvements in ‘Work organization and job content’ and ‘Interpersonal relations and leadership’. The concurrent deterioration in both ‘Role conflicts’ and ‘Social support’ from supervisors and colleagues may be related to the fact that the Lean-inspired ‘Problem Solving’ activity failed at this unit and the social support among the employees could not be promoted [26].

Due to the influence of multiple, concurrent changes (‘confounders’) and the heterogeneity in the Lean applications, a causal relationship between Lean and psychosocial work factors is difficult to establish. This has drawn the attention of researchers towards studying the changes in psychosocial work factors in the light of how and in which context Lean is implemented [8]. In the present study, we strove to take an approach inspired by realistic evaluation methods [27] by incorporating such information into the analysis. We did this by using a synthesis of the Lean literature [1, 5, 8, 23–25] about the psychosocial work environment, qualitative data in context and Lean implementations to predict expected changes in psychosocial factors. A significant regression analysis between expected and actual results in all three units showed that the Lean literature synthesis and the qualitative information we had about the units were very helpful in foreseeing the actual pattern of changes. Some of the scales, which were predicted to be most relevant to Lean, implementation also behaved quite in line with Lean intervention results. However, the ‘Vertical trust’ scale was an exception which behaved oppositely. Even if this is not in line with what was predicted, it does seem to be in line with the leadership changes which occurred in the units. For example, the leadership change on Ward-II before T2, which was welcomed by the staff, could have caused an increase in ‘Vertical trust’. This could be one way of predicting change patterns in the psychosocial work environment caused by Lean intervention that are testable with a combination of quantitative and qualitative data. It shows the usefulness of combining theory and data to understand intervention impacts in a complex and changing reality.

In summary, the findings from the three settings are in line with previous research and suggest that Lean may be related to improved psychosocial work conditions, particularly when a high degree of participation is a central part [5, 7, 12]. Particularly, it adds to the literature by showing that issues relating to work organization, job content, interpersonal relations and leadership seem to be related to changes in Lean implementation. However, the findings from this study do not show any consistent pattern in how Lean is related to ‘Demands at work’ and ‘Values at the workplace’.

### Strengths and limitations

A longitudinal study like this study entails all the complexity of the real world, which also brings significant research challenges. The challenge of investigating the effects of an intervention on certain variables lies in separating the relationship between the intervention (in this case, Lean) and the variables (in this case, the psychosocial work environment) from other changes, e.g. changes in leadership which take place continuously in a real life setting. Instead of trying to control these issues through design or by statistics, we aimed to track and integrate them into the analysis. This was done by studying several units, by continuously gathering data on the implementation process and other changes taking place concurrently and using this data in this study in combination with the quantitative data. Concurrently, the three units ended up with three different, rather distinct, Lean implementation patterns: one full implementation, one partial implementation and one deteriorating implementation. This allowed us to compare and contrast the interventions themselves and the corresponding psychosocial work environment outcomes, mimicking a dose–response relationship.

A comprehensive survey using a psychometrically validated questionnaire addressing the psychosocial work environment in a health care setting at two points in time during the Lean implementation process is the major strength of this study. The analysis is based on longitudinal data which is advantageous for investigating relationships between different variables [28]. A pure ‘before and after’ design was not possible as we gained access to the empirical settings only after Lean implementation had started. The fact, that T1 is not a clean measure of the situation before the Lean implementation, warrants caution in interpreting the findings as effects of Lean. However, it may also be argued that the mere concept of a baseline is an anomaly in organizational research of complex change, as there may not be a clear starting point for the intervention, and multiple changes happen simultaneously and continuously all the time. Although this design is not appropriate for conclusions about efficacy or for making causal inferences, it is helpful for understanding how a Lean implementation can relate to psychosocial work environment changes in the real world.

Using data solely based on personal statements and reports may lead to ‘common method bias’ [29] which may be avoided by using other data sources in addition to self-reports [28]. In this study, we incorporated qualitative information from observations, documentation and interviews into the analysis in the form of testable expected outcomes which might be a form of ‘multi-source assessment’ [30].

Survey response rate, although only one important factor among other issues, e.g. poor questions, inappropriate response scales etc., is mostly considered a big issue in health care studies [31]. The response rates in this study were 58% and 62% at T1 and T2 respectively which are not unusual response rates. A general issue with response rate is that those who answer surveys are often considered to be those who have more interest in the matter under consideration [32]. However, the low response rates may not necessarily be related to excessive levels of response bias for homogenous professional groups, in this case, nurses [33]. According to the qualitative data, some employees on one ward in this study (Ward-II) left between T1 and T2 because they were unhappy with the changes relating to Lean. Therefore, regarding this ward, there is a risk that those who were happier with the Lean changes constituted a larger share of potential respondents at T2. However, although the impact of this potential bias on the survey results remains unclear, it should be noted that the survey measured psychosocial work factors, not Lean as such. Also, at least according to the qualitative information, this potential bias seems to be limited to one ward (Ward-II), and not the ward with the greatest improvements (Ward-I).

The results in this study are limited to nurses’ and nurse aides’ perceptions of the psychosocial work environment. In future, including physicians in a study with a similar aim regarding the psychosocial work environment would be an important contribution.

## Conclusions

The content of a Lean intervention, its implementation and the context in which it is implemented may have a collective effect on the psychosocial work environment which is very difficult to capture. We tried to track a Lean footprint on the psychosocial work environment by combining longitudinal data on psychosocial work factors, multi-source data on Lean implementation and context. At the very least, we were able to capture a clear contrast between the footprints on Ward-I and ED which had opposite Lean implementation processes. Thus, although the design of this study does not allow causal inferences, the study does suggest that Lean interventions may be related to changes in the psychosocial work environment; positively when Lean is fully implemented and negatively when Lean changes deteriorate. Specifically, this study adds to the literature by suggesting that Lean implementation may be particularly relevant in relation to the ‘Work organization and job content’ and ‘Interpersonal relations and leadership’ domains of the psychosocial work environment. Also, health care managers should note that a failed implementation may actually worsen the work environment.

## Authors’ information

Waqar Ulhassan, PhD is a researcher at Medical Management Center, Karolinska Institute. His research interests focus on the implications of quality improvement programs on health care employees’ working and well-being, in particular teamwork and psychosocial work environment.

Ulrica von Thiele Schwarz is a psychologist and an associate professor in psychology. She is currently working at Medical Management Centre, Karolinska Institutet and the Department of Psychology, Stockholm University. Her main research theme is applying a behavioral perspective to interventions and continuous improvement within the work setting. Overall, the research is concerned with further the understanding on effective management of change in the workplace setting, and links between work conditions, health productivity and quality.

Johan Thor, MD, MPH, PhD, Director and lecturer; the Jönköping Academy for Improvement of Health and Welfare, Jönköping University (Sweden); research associate, the Medical Management Centre, Karolinska Institutet. Dr. Thor has devoted his career to promoting, and studying, healthcare improvement, covering process improvement, lean thinking, patient involvement, evidence-based medicine, and patient safety.

Hugo Westerlund is Professor of Epidemiology at the Stress Research Institute, Stockholm University. One of his main research interests is the impact of work, work organization, and work environment on the health and well-being of the employees. He mainly works with large cohort studies but has also conducted qualitative research.

## Electronic supplementary material

Additional file 1: **Graphical comparison of results at three settings.** Changes from first to follow-up measurement one and a half year apart (differential scores) in ratings of psychosocial work environment factors in the three settings. (PDF 8 KB)
