# Supplementary figures and images for: Interactions between lean management and the psychosocial work environment in a hospital setting – a multi-method study
Source: BMC Health Serv Res. 2014 Oct 22;14:480. doi: 10.1186/1472-6963-14-480 (PMC4282497; doi:10.1186/1472-6963-14-480)

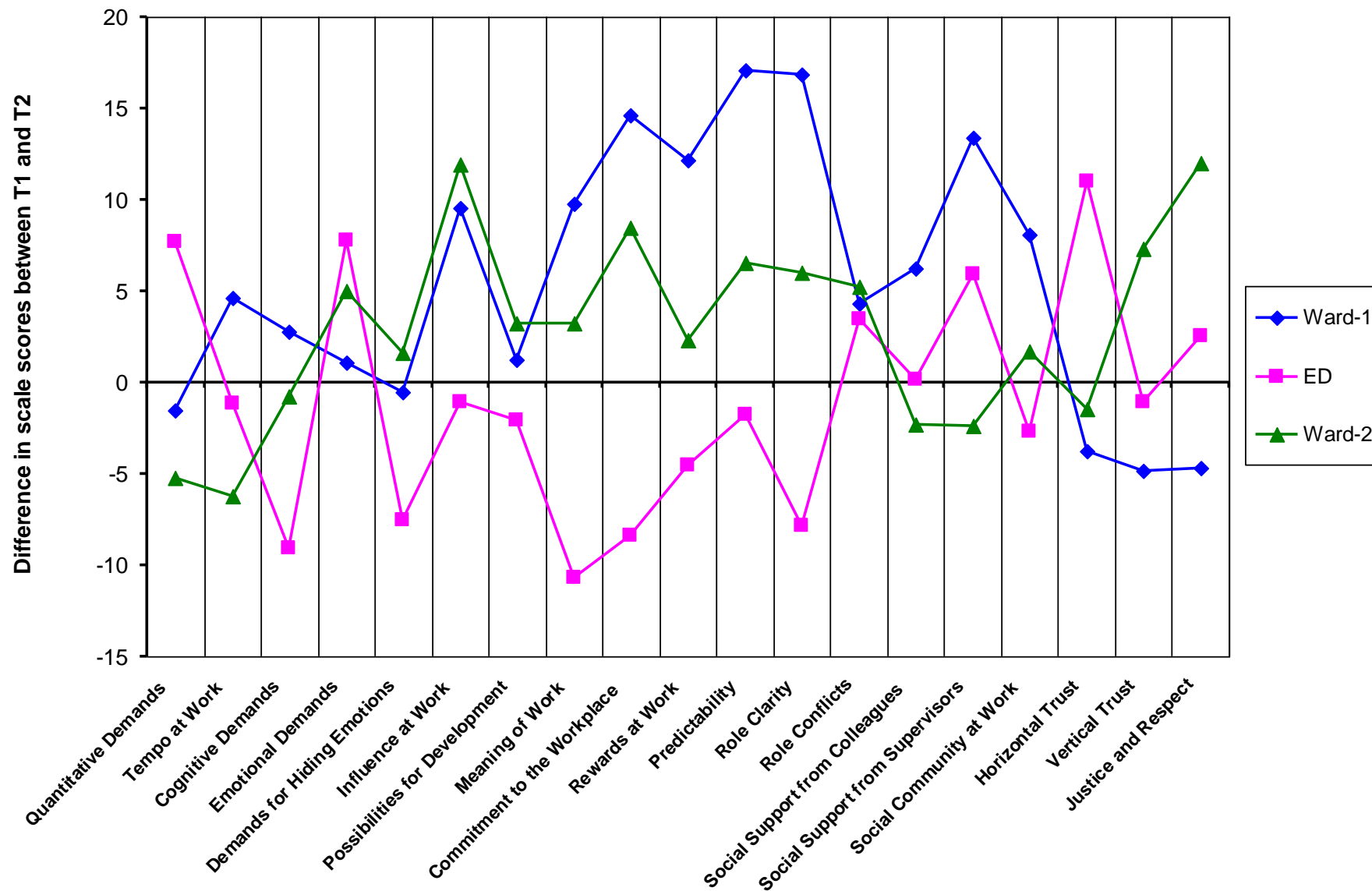

Supplement: Supplementary file 1 — Additional file 1: Graphical comparison of results at three settings. Changes from first to follow-up measurement one and a half year apart (differential scores) in ratings of psychosocial work environment factors in the three settings. (PDF 8 KB) [file 12913_2014_3580_MOESM1_ESM.pdf]
